# Reflections on the Brain Conference 2024

**DOI:** 10.1093/braincomms/fcae118

**Published:** 2024-05-03

**Authors:** Manuela Marescotti, Laurent Sheybani

**Affiliations:** Edinburgh, UK; London, UK

## Abstract

**Graphical abstract:**
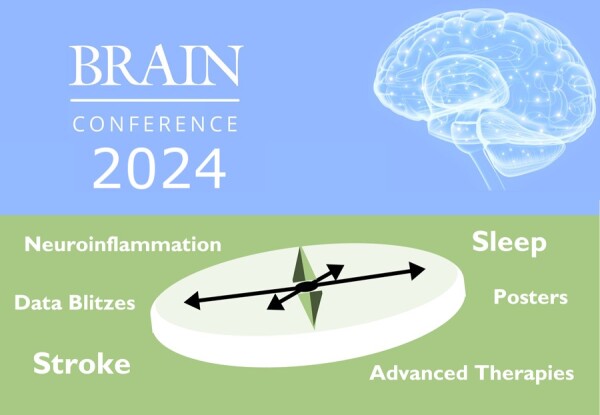

Welcome to Volume 6 Issue 3 of *Brain Communications*. In this editorial, we report the highlights of the Brain Conference 2024 (https://conference.guarantorsofbrain.org/), and we announce the winner of the *Brain Communications* Early Career Researcher Award 2023, for which nominations were invited in January 2024.^1^

This year, the Brain Conference was held in London on March 15. It was the first ever in-person Brain Conference and gave attendees the opportunity to attend high-quality talks, covering various aspects of neuroscience and neurology, with time for more informal discussions with leading scientists in their fields. The programme was designed to group talks by topic sessions (sleep, advanced therapies, neuroinflammation and stroke), and each session’s Chair was involved in the selection of speakers. The organizers also reserved time for a ‘Data Blitzes’ session where authors of outstanding abstracts had the opportunity to present their work in 5 minutes. Moreover, each Data Blitzes speaker presented their work during the poster session.

One of the Guarantors of Brain’s missions is education and in line with this, Professor Colin Espie (University of Oxford) was invited to give a plenary lecture on sleep. Professor Espie addressed the main characteristics of the sleep–wake cycle, insisting on its highly dynamic nature and relevance for human health. A particularly important aspect, which was also discussed in Professor Vyazovskiy’s talk, is the concept of local sleep, which describes the fact that one specific brain region can show signs of sleep, while the rest of the brain is fully awake. Professor Espie then illustrated selected sleep disorders, gently leading the audience towards the talks of the Sleep session presented by Professor Vyazovskiy (University of Oxford), Professor Mormann (Bonn University) and Dr Barateau (Montpellier University).

During the ‘Advanced Therapies’ session, Professor Gabriele Lignani (UCL) presented an elegant approach where gene therapy is used to treat cases of pharmacoresistant epilepsy,^2^ such as those consecutive to focal cortical dysplasia, a leading cause of drug-resistant epilepsy.^3^ The procedure has been tested so far in mice and human organoids. Lignani and team designed a transgene construct that selectively translates into a potassium channel in neurons presenting with excessive activity. This selective inactivation of hyperexcitable neurons is thus a promising therapeutic approach to deliver a targeted silencing of cells recruited in epileptic activity.

In the same ‘Advanced Therapies’ session, Dr Catherine Mummery (UCL) presented the current state of research related to therapies targeting specific proteins involved in Alzheimer’s and other neurocognitive diseases. Dr Mummery's work is involved in early-phase trials, where therapy candidates are given to patients for the first time. These therapies act on reducing levels of proteins identified as central to the biology of specific diseases. Monoclonal antibodies and gene silencing are two examples of promising therapies studied by Catherine Mummery’s team.

Within the context of Data Blitzes, our Editor-in-Chief, Professor Tara Spires-Jones (University of Edinburgh) announced the *Brain Communications* early Career Researcher award 2023 winner, Dr Stanislau Hrybouski (University of Pennsylvania). This is the second year this annual prize has been awarded to the first author of an outstanding paper published in our journal during the previous year. Dr Hrybouski had the opportunity to give a talk about his paper ‘Aging and Alzheimer’s disease have dissociable effects on local and regional medial temporal lobe connectivity’.^4^ In his work, Dr Hrybouski studied functional connectivity in the mesial temporal lobe in normal aging and patients with Alzheimer’s disease, guided by the known topographical sequence of tau deposition in this brain area. Hyperconnectivity has been associated with tau accumulation, and in line with early tau deposition in the anterior mesial temporal lobe, he found increased connectivity of anterior mesial temporal lobe in cognitively unimpaired subjects who were positive for an Alzheimer’s disease biomarker (Aβ+). Contrasting with this, in cognitively impaired patients, anterior connectivity was decreased in comparison with Aβ+ unimpaired subjects. This is in line with the expected ‘circuit breakdown’ in this population affected by cognitive deficits. Conversely, intra-mesial temporal lobe connectivity was reduced in normal aging. The inverted U-shape change in intra-mesial temporal lobe connectivity in Aβ+, cognitively unimpaired patients (increased connectivity) and Aβ+, cognitively impaired patients (decreased connectivity), as well as the contrasting pattern of overall decrease in normal aging, improve our knowledge of connectivity in a core brain region of Alzheimer’s disease physiopathology.

In the ‘Neuroinflammation’ session, Professor Astrid Iversen (University of Oxford), presented very recent results from an international study that she co-led with Professor Lawson (University of Bristol), Professor Fugger (University of Oxford and Aarhus University) and Professor Willerslev (University of Cambridge).^5^ Using genetic analyses from up to 10 000-year-old genomes across Europe and Central Asia, their work suggests a central role of pastoralist ancestry in shaping the genetic risk, and geographical pattern, of multiple sclerosis. They show that a specific genetic variant, known to be associated with an increased risk of multiple sclerosis, underwent a positive selection in steppe pastoralist populations. While the reason for this positive selection remains unclear, a heightened protection against infection could be a cause, as suggested by the authors.

The poster prize, selected by the Guarantors of Brain, was awarded to Dr Patel Sahil (UCL) for his work on G-protein coupled receptor activated by non-prescription agent. Designer receptor exclusively activated by designer drugs can selectively modulate the activity of neurons, which could be highly relevant for various neurological conditions. However, clozapine, which is currently used to activate designer receptor exclusively activated by designer drugs, has many side effects (e.g. lowered seizure threshold, neutropenia) that limit clinical translation. Hence, they engineered a new version of the muscarinic receptor hM4Di (the original target of clozapine) that is sensitive to diphenydramine, which has less side effect. The authors argue that the clinical translation is thus decreased thanks to this reduced side effect profile.

The Brain Conference is the result of the joined forces of Guarantors of Brain, their fellows and their journal (*Brain* and *Brain Communications*) teams. This exciting event illustrated the virtuous cycle between the high-quality science published by our journals, the fellowships and events funded through the income of these journals, and eventually the cutting-edge science delivered by these funded projects.^6^ The great science we heard about on this occasion is of inspiration for researchers doing their hard work every day, and we hope to make it grow over subsequent years. We are deeply grateful to our pro-active audience and speakers: Colin Espie, Vladyslav Vyazovskiy, Florian Mormann, Lucie Barateau, Gabriele Lignani, Matthis Synofzik, Catherine Mummery, David Hunt, Arman Eshaghi, Astrid Iversen, Steffen Tiedt, Zahraa Al-Ahmady, Hanna Willis and all young(er) speakers of the Data Blitzes.

The cover of this issue shows an artistic representation of a female scientist working in an epilepsy lab, utilizing zebrafish and electrophysiology, along with other techniques, to investigate therapies for childhood genetic epilepsies, courtesy of Paige Whyte-Fagundes *et al*.^7^
